# In Vitro–In Vivo Correlations Based on In Vitro Dissolution of Parent Drug Diltiazem and Pharmacokinetics of Its Metabolite

**DOI:** 10.3390/pharmaceutics11070344

**Published:** 2019-07-16

**Authors:** Constantin Mircioiu, Valentina Anuta, Ion Mircioiu, Adrian Nicolescu, Nikoletta Fotaki

**Affiliations:** 1 Department of Applied Mathematics and Biostatistics, Faculty of Pharmacy, “Carol Davila” University of Medicine and Pharmacy, 020956 Bucharest, Romania; 2 Department of Physical and Colloidal Chemistry, Faculty of Pharmacy, “Carol Davila” University of Medicine and Pharmacy, 020956 Bucharest, Romania; 3 Department of Biopharmacy and Pharmacokinetics, Titu Maiorescu University, 004051 Bucharest, Romania; 4 Department of Medicine, Queen’s University, Kingston, ON K7L 3N6, Canada; 5 Department of Pharmacy and Pharmacology, University of Bath, Claverton Down, Bath BA2 7AY, UK

**Keywords:** in vitro–in vivo correlation (IVIVC), diltiazem, mathematical modeling, metabolites, dissolution

## Abstract

In this study a novel type of in vitro–in vivo correlation (IVIVC) is proposed: The correlation of the in vitro parent drug dissolution data with the in vivo pharmacokinetic data of drug’s metabolite after the oral administration of the parent drug. The pharmacokinetic data for the parent drug diltiazem (DTZ) and its desacetyl diltiazem metabolite (DTZM) were obtained from an in vivo study performed in 19 healthy volunteers. The pharmacokinetics of the parent drug and its metabolite followed a pseudomono-compartmental model and deconvolution of the DTZ or DTZM plasma concentration profiles was performed with a Wagner–Nelson-type equation. The calculated in vivo absorption fractions were correlated with the in vitro DTZ dissolution data obtained with USP 2 apparatus. A linear IVIVC was obtained for both DTZ and DTZM, with a better correlation observed for the case of the metabolite. This type of correlation of the in vitro data of the parent compound with the in vivo data of the metabolite could be useful for the development of drugs with active metabolites and prodrugs.

## 1. Introduction

One of the goals of in vitro–in vivo correlations (IVIVCs) is the estimation of the in vivo release of an active substance from orally administered pharmaceutical formulations based on in vitro dissolution data. For extended release formulations, reasonable linear correlations have been obtained from a number of IVIVC studies [1,2,3,4,5]. In other cases, linear correlations were not satisfactory [6,7,8,9], or the data could not be correlated [2,10,11]. Although in the case of extended release formulations linear relationships are frequently obtained, both the United States Pharmacopoeia (USP) and the Food and Drug Administration (FDA) state that non-linear models are also acceptable to describe IVIVCs [12,13,14]. Non-linear models have been proposed by Polli et al. and Dunne et al. [15,16,17]. Model-independent methods are commonly used for the development of IVIVCs, but model-dependent methods are also applied [15,16,17,18,19,20]. For example, Dunne et al. started from survival curve methods considering the time at which a drug enters the solution (in vitro or in vivo) as a random variable and correlated the in vitro and in vivo parameters postulating different relations between distribution functions or their probability densities [16,17]. The distribution functions were obtained by cumulative dissolution and the probability densities were determined by the rate at which the drug is released from the pharmaceutical formulation. These non-linear models were considered as scientifically sound IVIVCs that contrast with the “empirical” non-linear functions such as the sigmoid, Weibull, Higuchi, or Hixson–Crowell methods [21]. All these models remain essentially empirical, and the more complex the model is the more unstable is the fitting algorithm and the risk of non-uniqueness of the solutions [22,23].

Diltiazem (DTZ), a benzothiazepine calcium channel blocker, has been widely used in the treatment of stable, variant, and unstable angina pectoris, systemic hypertension, and supraventricular tachycardias [24,25]. It is subjected to extensive and highly variable hepatic first-pass metabolism by CYP3A4, and only 2%–4% of the unchanged drug is excreted in the urine. The pharmacokinetics of DTZ in healthy volunteers indicated a biphasic elimination with a distribution half-life of 0.3 ± 0.2 h, an elimination half-life of 3.1 ± 1 h and an apparent volume of distribution of 5.3 ± 1.7 L/kg after intravenous (i.v.) administration of 15 mg, and an elimination half-life of 3.2 ± 1.3 h after oral administration of 60 mg [26]. The absolute bioavailability of DTZ ranged from 24% to 74% (mean 42% ± 18%), and the inter-individual variability may be explained by the highly variable first-pass effect [24]. Non-linearity and a slight increase of the half-life were observed when the dose was increased. Notably, in young healthy volunteers DTZ did not show linear kinetics between single and multiple doses [27].

DTZ has several metabolites in humans: Desacetyl DTZ (DTZM), N-monodesmethyl DTZ, desacetyl N-monodesmethyl DTZ, and desacetyl DTZ N-oxide, with average maximum plasma concentrations of 10%, 15%, 26%, and 13%, respectively, of the mean maximum DTZ concentration [28]. Therefore, the analysis of the pharmacokinetics of DTZ and its metabolites, as well as the development of IVIVCs can be useful in improving the general administration schedule, the personalization of therapy and the development of DTZ formulations for oral administration.

The aim of this study was to develop IVIVCs for orally administered DTZ formulations. Apart from the traditional IVIVC approach in which the in vitro drug dissolution is correlated with its in vivo absorption calculated by deconvolution, a novel method was developed. The in vitro dissolution of DTZ was correlated to the in vivo absorption estimated by a deconvolution method that uses the pharmacokinetic data of the active metabolite (DTZM). This approach could be valuable for the development of IVIVCs for drugs with significant plasma levels of metabolites.

## 2. Materials and Methods

### 2.1. Chemicals and Reagents

Diltiazem hydrochloride (batch 4) and haloperidol (batch 1) reference standards were purchased from the European Directorate for the Quality of Medicines (EDQM, Strasbourg, France), while desacetyl diltiazem hydrochloride (batch JOC143) was obtained from the United States Pharmacopeia (USP, Rockville, MD, USA). Cardiazem^®^ 60 mg tablets (Hoechst Marion Roussel, batch number 1099841) were used for the in vitro and in vivo studies.

HPLC gradient grade acetonitrile and methanol were purchased from Merck KGaA (Darmstadt, Germany), whereas HPLC grade methyl tert-butyl ether was acquired from Sigma-Aldrich (Taufkirchen, Germany). All other reagents were of analytical grade and used without further purification. Ultrapure water (resistivity 18.2 MΩ·cm at 25 °C, Total Organic Carbon (TOC) < 5 ppb) was obtained from a Milli-Q (Millipore, Milford, MA, USA) water purification system. Blank human plasma was obtained from the Army Centre of Transfusion Hematology (Bucharest, Romania).

### 2.2. In Vitro Dissolution Studies

Dissolution studies of Cardiazem^®^ 60 mg tablets were performed using a USP 2 dissolution apparatus (DT 800 Erweka GmbH, Heusenstamm, Germany) at 75 rpm. The dissolution medium (ultrapure water, 900 mL) was deaerated and maintained at 37 ± 0.5 °C. Aliquots of 5 ± 0.1 mL were withdrawn at 10, 15, 20, 30, 60, 120, and 180 min, and immediately replaced with an equal volume of fresh medium maintained at the same temperature. The samples were filtered through a 0.45 μm Teflon^®^ filter, and the drug concentrations were determined by measuring the absorbance of each sample at 237 nm on a V-530 UV-VIS spectrophotometer (JASCO Ltd., Tokio, Japan). Diltiazem concentrations were calculated from linear calibration curves. Dissolution data for each compound are reported as mean values of 12 replicates, and the coefficient of variation (CV%; [mean value/standard deviation] × 100%) was calculated.

### 2.3. Clinical Study

In vivo data were obtained in a pharmacokinetic study after administration of two 60 mg Cardiazem^®^ tablets to 19 healthy volunteers in a single-dose study under fasting conditions.

The study was carried out in accordance with the basic principles defined in the Helsinki Declaration of 1964 as revised in 2013, as well as with the International Conference on Harmonization (ICH) Good Clinical Practice regulations. The study was conducted at the National Institute for Aeronautical and Space Medicine «Gen. Dr. Av. Victor Atanasiu» within the Central Clinical Emergency Military Hospital in Bucharest (Romania).

The study protocol (protocol code: DILTZARE155/2002) was approved by the Institutional Ethics Committee of Biopharmacy & Pharmacol Res S.A. (approval number 32, 4 July 2007), as well as by the Romanian National Agency for Medicines and Medical Devices (approval number 337, 24 July 2007).

The study subjects (*n* = 19) were of Caucasian race, aged between 19 and 30 years (24.3 ± 3.34) and with a body mass index between 19 and 27 (22.43 ± 1.87). All subjects were healthy according to their medical and social history, physical examination, and laboratory tests. The subjects had no history of drug or alcohol abuse, hypersensitivity to the investigational products, and did not take any medication for two weeks before dosing. Alcohol, tobacco, as well as caffeine containing beverages were forbidden for 48 h before as well as during the study. All subjects gave written informed consent prior to study enrolment and were allowed to terminate their participation in the trial at any time, without restrictions. Standard meals were provided to the subjects at four and nine hours after drug administration.

Venous blood samples (5 mL) were collected into heparinized tubes through a catheter inserted in the antecubital vein before (time 0) and at 0.5, 1, 1.5, 2, 2.5, 3, 3.5, 4, 5, 6, 7, 8, 10, 12, and 24 h after drug administration. Blood samples were centrifuged at 5 °C for six minutes at approx. 3000 rpm. Plasma was separated in two equal aliquots (1.2–1.3 mL), transferred to labeled 1.5 mL polypropylene tubes and immediately frozen and stored at a < −20 °C until analysis.

### 2.4. Sample Treatment

Plasma samples (500 μL) were transferred to 10 mL disposable polypropylene tubes, to which 100 µL internal standard (IS) solution (containing 10 μg/mL haloperidol in methanol), 200 µL 0.2 M dipotassium phosphate buffer pH = 9 and 3 mL methyl *tert*-butyl ether were added. The tubes were vortex-mixed for 10 min and then centrifuged for 10 min at 4000 rpm. Of the organic layer 2.5 mL were removed and extracted with 200 µL 0.025 M phosphoric acid solution. After shaking for 10 min and centrifugation for 10 min at 4000 rpm, 25 μL of the acidic aqueous phase were analyzed by HPLC.

### 2.5. Preparation of Standard Solutions and Quality Control Samples

The stock solutions of DTZ and DTZM were prepared by dissolving an appropriate amount of each reference standard in methanol to yield concentrations of 100 µg/mL and 50 µg/mL, respectively, and serially diluted with the same solvent. 10 μL of the diluted solutions of each analyte were spiked into 80 μL of blank plasma, in order to obtain the calibration standard solutions with final concentrations of 2.5, 5, 10, 25, 50, 100, 250, and 500 ng/mL for DTZ and 1.25, 2.5, 5, 12.5, 25, 50 125, and 250 ng/mL for DTZM.

Quality control (QC) samples were prepared similarly, in order to obtain concentrations at the lower limit of quantification (LLOQ; 2.5 ng/mL for DTZ and 1.25 ng/mL for DTZM), and at low (QC_low_ = 7.5 ng/mL DTZ and 3.75 ng/mL DTZM), medium (QC_med_ = 150 ng/mL DTZ and 75 ng/mL DTZM), and high (QC_high_ = 300 ng/mL DTZ and 150 ng/mL DTZM) concentration levels.

All stock and standard solutions were protected from light and stored at −20 °C until use.

### 2.6. Chromatographic Analysis

Chromatographic analyses were performed on a Waters liquid chromatographic system (Milford, MA 01757, USA) consisting of a quaternary gradient system (600E Multisolvent Delivery System), in line degasser (Waters model AF), UV tunable absorbance detector (Waters model 486), and auto sampler (Waters model 717 plus). Empower Pro software (Waters, Milford, MA 01757, USA) was used to control the system, acquire and process data. The UV detector was set at 235 nm. The chromatographic separation was achieved on an Ascentis 5C18, 5-μm 150 × 2.1 mm column (Supelco, Bellefonte, PA 16823, USA) at a constant temperature (35 °C). The mobile phase consisted of an isocratic mixture of 0.025 M potassium di-hydrogen phosphate buffer containing 0.2% triethylamine adjusted to pH 2.2 and acetonitrile in a 72:28 (*v*/*v*) ratio, and delivered at 0.35 mL/min flow rate. Of each sample 25 µL were injected into the chromatographic column.

Method validation was performed in accordance with the bioanalytical method validation guidelines of the FDA, including selectivity, linearity, limits of quantification, accuracy, precision, recovery, dilution effects, and stability [29]. Assay specificity was evaluated in relation to interferences from the endogenous matrix components of drug free plasma samples of six different origins. The calibration curves of both DTZ and DTZM were constructed by plotting DTZ or DTZM to IS peak area ratios versus concentration (ng/mL), using data obtained from triplicate analysis of the calibration standard solution (in the range 2.5–500 ng/mL for DTZ and 1.25–250 ng/mL for DTZM). The lower limit of quantification (LLOQ) was set as the lowest concentration on the calibration curve. Within-run and between runs precision and accuracy were estimated by analyzing five replicates of the LLOQ and the QC samples in a single analytical run and on five consecutive days, respectively. The acceptance criteria for precision and accuracy were: Relative Standard Deviation (RSD)% ≤15% and bias within ±15% for the QC samples and RSD% ≤20% and bias within ±20% for the LLOQ samples. The absolute recovery of DTZ and DTZM was determined using five replicates of the three concentration level QC samples. Bench-top, extract, stock solution, freeze-and-thaw, long-term, and post-preparative stability studies were also performed to evaluate the stability of both DTZ and DTZM.

### 2.7. Treatment of In Vivo Data

Estimation of pharmacokinetic parameters of the in vivo parameters by non-compartmental analysis was performed using subroutines of the KINETICA 4.2 software (Innaphase Corp, Philadelphia, PA 19102, USA). The maximum concentration (C_max_) and the corresponding peak times (T_max_) were determined from the individual drug plasma concentration-time profiles. The elimination rate constant (*k_e_*) was obtained from the least-square fitted terminal log-linear portion of the plasma concentration–time profile. The elimination half-life (t_1/2_) was calculated as 0.693/*k_e_*. The area under the curve to the last measurable concentration (AUC_0–t_) was calculated by the trapezoidal rule method. The area under the curve extrapolated to infinity (AUC_0–∞_) was calculated as AUC_0–t_ + *C_t_*/*k_e_*, where *C_t_* is the last measurable concentration. Shapiro–Wilk statistic W-test (SW–W) was used to evaluate normality of data distribution, with a *p* < 0.05 set as threshold for statistical significance.

Compartmental analysis of the in vivo data was performed using TOPFIT 2.0 software (Thomae GmbH, Germany). Fitting performance was assessed based on the Akaike (AIC) and Schwarz (SC) criteria (both based on the sum of “errors” corrected by a “penalty” function proportional to the number of parameters model: *AIC* = *N·lnSS* + 2*p*; *SC* = *N·lnSS* + *p·lnN*) [22], where *N* is the sample size (i.e., number of data points), *p* represents the number of model parameters and *SS* is the sum of squares error.

The significance of the differences in the fitting performance of two nested models, a more complicated one (with *p* parameters) and a simpler one (with *q* parameters, *q* < *p*), was evaluated by comparing the relative increase in the sum of squares $\left(\frac{SS_{q}-SS_{p}}{SS_{p}}\right)$ with the relative decrease in degrees of freedom $\left(\frac{df_{q}-df_{p}}{df_{p}}\right)$ going from the more complicated to the simpler model, based on the F ratio:

$$F=\frac{SS_{q}-SS_{p}}{SS_{p}}\frac{df_{p}}{df_{q}-df_{p}}$$

where the degrees of freedom *df* equals the difference between the sample size *N* and the number of parameters of each model ($df_{p}=N-p$ and $df_{q}=N-q$, respectively).

A model is considered more efficient if it is simple, has a minimum number of parameters, is “phenomenologically” justified and errors are comparable with experimental errors and physiological variability.

### 2.8. Model-Independent Estimation of In Vivo Absorption/Dissolution by the Deconvolution of In Vivo Pharmacokinetics

The fraction of drug absorbed was calculated using the Wagner–Nelson equation [30]:

$$FRA\left(t_{i}\right)=\frac{{c_{p}}_{d}\left(t_{i}\right)+\int_{0}^{t_{i}}k_{e}c_{pd}dt}{\int_{0}^{\infty }k_{e}c_{pd}dt}=\frac{{c_{p}}_{d}\left(t_{i}\right)+k_{e}AUC{\left(PD\right)}_{0-t_{i}}}{k_{e}AUC{\left(PD\right)}_{0-\infty }},$$

where, *FRA*(*t_i_*), fraction of the drug absorbed at time $t_{i}$; ${c_{p}}_{d}\left(t_{i}\right)$, plasma concentration of the parent drug at time $t_{i}$; $k_{e}$, elimination rate constant for the parent drug; $AUC{\left(PD\right)}_{0-t_{i}}$, area under the concentration–time curve of the parent drug from time 0 to time *t_i_*; $AUC{\left(PD\right)}_{0-\infty }$, area under the concentration–time curve of the parent drug from time 0 to infinity.

In the case of metabolites, a Wagner–Nelson type equation was applied for the calculation of the apparent fraction of the metabolized drug (FRM):

$$FRM\left(t_{i}\right)=\frac{c_{m}\left(t_{i}\right)+\int_{0}^{t_{i}}k_{e}^{m}c_{m}dt}{\int_{0}^{\infty }k_{e}^{m}c_{m}dt}=\frac{c_{m}\left(t_{i}\right)+k_{e}^{m}{\left[AUC-M\right]}_{0-t_{i}}}{k_{e}^{m}{\left[AUC-M\right]}_{0-\infty }},$$

where, *FRM*(*t_i_*), fraction of the metabolized drug at time $t_{i}$; $c_{m}\left(t_{i}\right)$, plasma concentration of the metabolite at time $t_{i}$; $k_{e}^{m}$, elimination rate constant of the metabolite.

The elimination rate constant was estimated by performing both a non-compartmental analysis (linear regression of the last points of the logarithmic data) and a one-compartmental modeling of the mean plasma levels.

If the absorption and metabolism can be assumed to be rapid, *FRM*(*t_i_*) could be considered an estimation of *FRA*(*t_i_*). Based on this assumption, an in vitro dissolution–in vivo metabolism correlation could be expected.

## 3. Results

### 3.1. In Vitro Dissolution of Diltiazem

**Figure 1:**
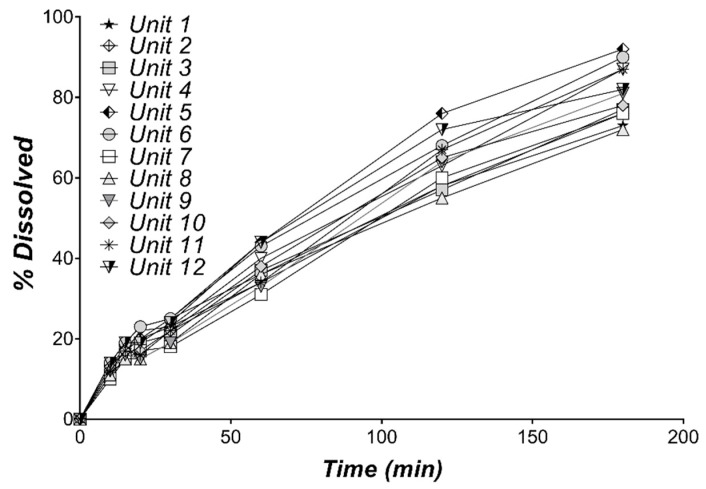
Individual in vitro dissolution profiles of DTZ from 60 mg Cardiazem^®^ tablets in 900 mL water, using USP Apparatus 2, at 75 rpm (*n* = 12).

The individual DTZ dissolution profiles in water are presented in Figure 1 and the mean % dissolved over time are shown in Table 1. A 80.92% DTZ dissolved is observed after 3 h. Since DTZ is lipophilic (logP 2.7) [31], its administration as a hydrochloride salt helps the rapid dissolution in the stomach. Its precipitation in the intestine is likely unavoidable (pKa = 8.06) and could account for its absorption variability. Based on the small variability of the in vitro dissolution profiles, as revealed by the low values of CV% (Table 1), the mean dissolution profile can be used for the development of IVIVCs.

### 3.2. Chromatographic Method Validation

**Figure 2:**
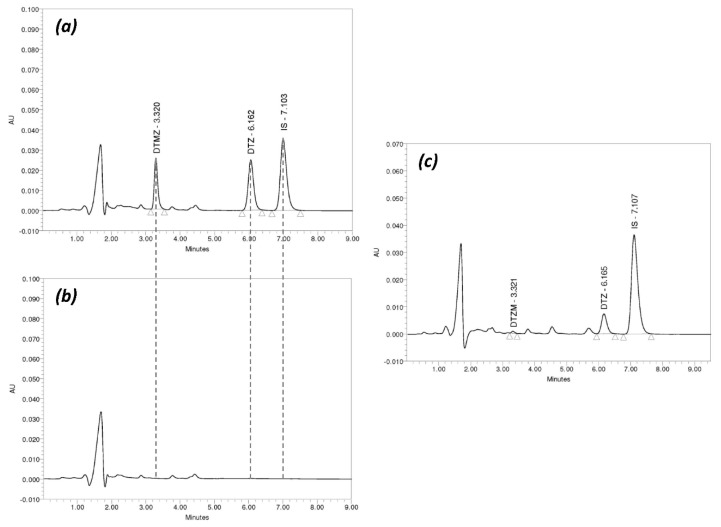
Typical HPLC chromatograms of DTZ and DTZM in plasma: (**a**) Chromatogram of a standard sample containing DTZ (500 ng/mL) and DTZM (250 ng/mL) and internal standard (IS); (**b**) chromatogram of blank plasma; (**c**) chromatogram of a plasma sample obtained from one on the study subjects two hours after drug administration (DTZ—154.4 ng/mL, DTZM—3.9 ng/mL).

No interference between the endogenous matrix components and DTZ or DTZM was observed, indicating selectivity of the HPLC method in the plasma samples (Figure 2). Calibration curves were linear over the concentration range 2.5–500 ng/mL for DTZ (Y = 1.21e–003X + 2.03e–003; R^2^ = 0.9997), and 1.25–250 ng/mL for DTZM (Y = 1.59e–003X + 2.33e–004; R^2^ = 0.9997).

The LLOQ was 2.5 ng/mL for DTZ and 1.25 ng/mL for DTZM, suggesting a good sensitivity of the analytical method.

Both within-run and between runs accuracy and precision were within the accepted limits (Table 2) for the LLOQ and all the QC samples. The within-run precision (RSD%) ranged between 1.19% and 5.71%, whereas accuracy (% bias versus nominal concentration) ranged between 0.25% and 3.76%; the between run precision (RSD%) was between 3.53% and 8.03% whereas the % bias versus the nominal concentration was lower than 6% (Table 2).

The mean absolute recovery in plasma was 105.19% ± 4.81% for DTZ, 96.52 ± 5.59 for DTZM and 91.11 ± 3.32 for the IS, indicating lack of interference from the sample preparation method. Dilution effect was not observed either for DTZ or DTZM by means of a five-fold dilution with blank plasma.

Both DTZ and DTZM were stable in plasma for 5 h at room temperature, for 27 h during the chromatographic analysis (placed in autosampler) and for 97 days at −20 °C. There was no observed degradation of the samples under three cycles of freezing and thawing.

Typical HPLC chromatograms of DTZ and DTZM in plasma are presented in Figure 2.

### 3.3. Pharmacokinetics of Diltiazem and Its Metabolite

**Figure 3:**
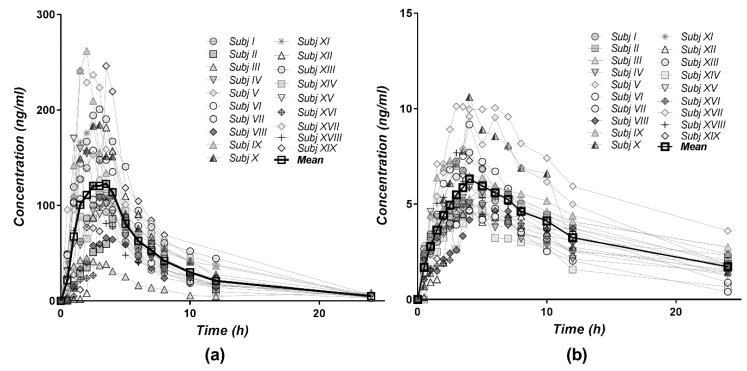
Individual and mean plasma concentration–time profiles for (**a**) DTZ and (**b**) DTZM after single dose oral administration of 120 mg diltiazem (2 × 60 mg Cardiazem^®^ tablets) to 19 healthy subjects.

The individual and mean plasma level profiles of DTZ and DTZM after oral administration of 120 mg of DTZ (two 60 mg Cardiazem^®^ tablets) were relatively homogenously distributed in the concentration–time space (Figure 3). The data could be interpreted as revealing three clusters, i.e., three volunteers with high, one with low plasma levels, and the remaining 15 volunteers having homogenously distributed profiles. The plasma concentrations of the metabolite DTZM were approximately 20 times lower than those of the parent drug.

**Figure 4:**
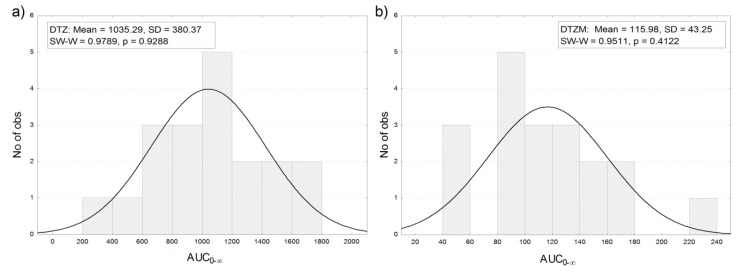
Frequency distribution of AUC_0–∞_ for (**a**) DTZ and (**b**) DTZM after the oral administration of 120 mg of DTZ in 19 healthy subjects (the observed significance level *p* of the Shapiro–Wilk statistic W (SW–W) indicates normality of the distribution (*p* < 0.05)).

The distribution of the areas under curves of the plasma concentration profiles after the administration of 120 mg of DTZ in the 19 healthy volunteers was approximately normal (Figure 4).

The summary of the main pharmacokinetic parameters of DTZ and DTZM in the 19 healthy volunteers, estimated by non-compartmental analysis, is presented in Table 3.

### 3.4. Compartmental Modeling of Diltiazem and Its Metabolite Pharmacokinetics

**Figure 5:**
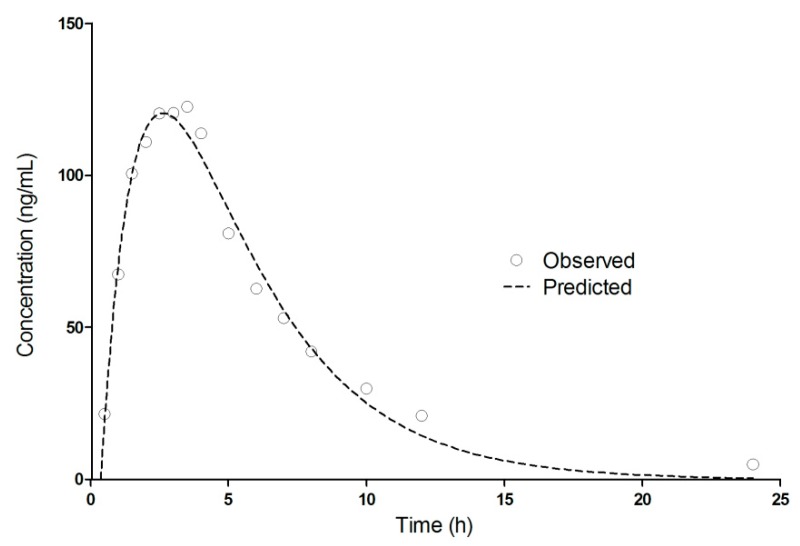
One-compartment pharmacokinetic modeling of DTZ mean plasma levels after oral administration of 120 mg DTZ (2 × 60 mg Cardiazem^®^ tablets) in 19 healthy subjects.

**Figure 6:**
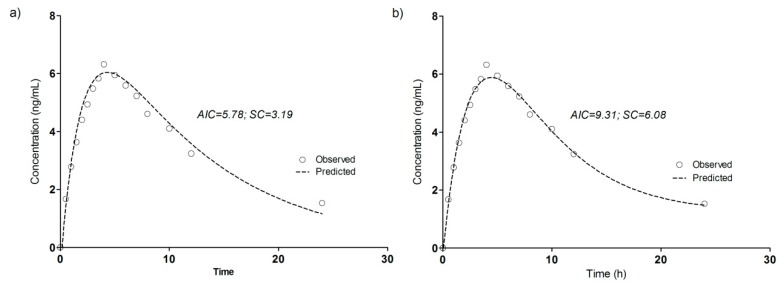
Pharmacokinetic modeling of DTZM mean plasma levels after oral administration of 120 mg DTZ (2 × 60 mg Cardiazem^®^ tablets) in 19 healthy subjects. (**a**) One-compartment model; (**b**) two-compartment model.

The pharmacokinetics of DTZ and DTZM were evaluated based on compartmental modeling. Based on work performed previously, it has been shown that the pharmacokinetics of the metabolites usually follow a pseudomono-compartmental model [32,33,34]. The pharmacokinetic modeling of DTZ and DTZM revealed that the mean plasma levels can be acceptably described by a one-compartment model after introducing a short lag-time (Figure 5). The use of the two compartmental model was just marginally better based on the Akaike and Schwarz criteria (Figure 6). Therefore, increase of the number of the parameters was not selected, as models with a high number of parameters are highly unstable, since small perturbations in the input data can lead to high differences in the solutions [23].

The derived pharmacokinetic parameters from the one compartmental modeling of the mean plasma levels of DTZ and DTZM after the oral administration of 120 mg DTZ are summarized in Table 4.

### 3.5. Model-Independent Estimation of In Vivo Absorption/Dissolution

**Figure 7:**
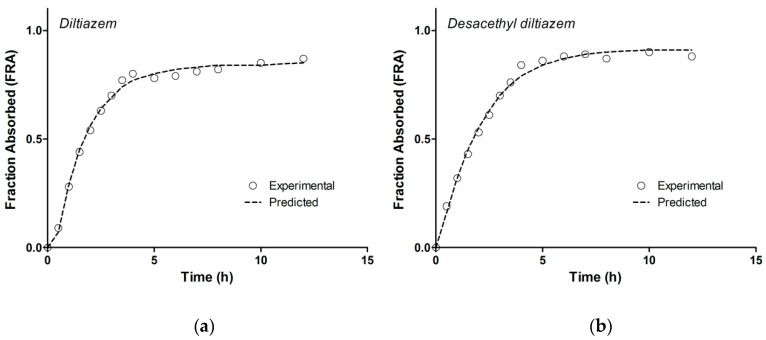
Fraction absorbed profiles calculated for (**a**) DTZ and (**b**) DTZM; observed: profiles calculated based on estimation of elimination rate constant with non-compartmental analysis, predicted: profiles calculated based on estimation of elimination rate constant with one-compartmental pharmacokinetic modeling.

The fractions of DTZ and DTZM absorbed over time as calculated by the Wagner–Nelson equations are presented in Figure 7. As the parent drug and metabolite follow a one-compartmental pharmacokinetic model (as presented in the previous section), this model independent deconvolution approach with the Wagner–Nelson method can be successfully applied to the in vivo DTZ and DTZM plasma concentration profiles.

The fraction absorbed profiles calculated based on estimation of elimination rate constant with both methods (non-compartmental analysis and one-compartmental pharmacokinetic modeling) were similar for DTZ and DTZM, revealing that the method used for the estimation of the elimination rate constant was robust. In the case of the DTZM, the elimination profile was simple and further metabolism was not observed. Furthermore, given the more polar character of DTZM compared to DTZ, its biliary excretion is less significant. Consequently, the variability of metabolite’s elimination constant should be lower than that of the parent drug. This is an important argument for using the metabolite plasma levels rather than those of the parent drug for estimating the in vivo dissolution of the parent drug. The results obtained by following a Wagner–Nelson approach reflect the combination of the in vivo dissolution, absorption and metabolism of the parent drug. In this chain of processes, the slowest process determines the overall result.

### 3.6. Correlation of Apparent Absorbed/Metabolized Fraction with In Vitro Dissolution of Diltiazem

**Figure 8:**
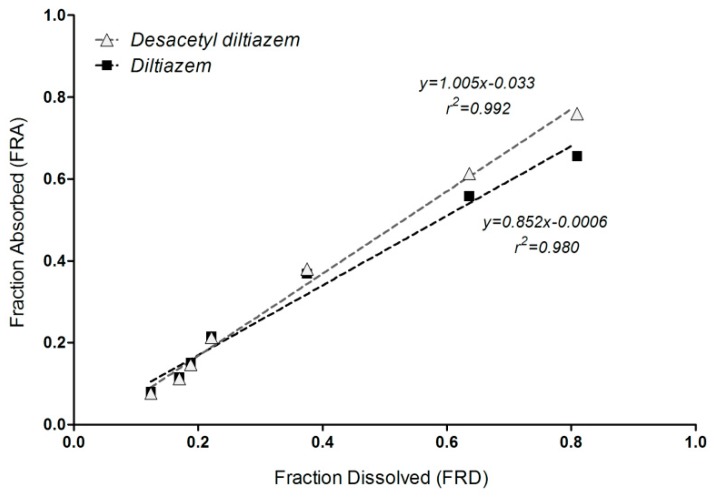
In vitro–in vivo correlation (IVIVC) model for DTZ (■) and DTZM (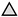).

Since it is not possible to estimate separately the in vivo dissolution, gastric empting, absorption, and metabolism of the parent drug, a mechanistic approach is not realistic. Therefore, an empirical approach was followed. The “apparent fraction absorption” calculated from plasma levels of DTZ and DTZM was correlated with the in vitro dissolution of DTZ. A Level A correlation between in vitro dissolution and estimated in vivo dissolution starting from the parent drug and its metabolite plasma level was achieved (Figure 8). Both correlations were linear, with correlation coefficients greater than 0.98.

The slope of the IVIVC model based on “metabolite pharmacokinetics—dissolution of parent drug” is close to 1, suggesting a superposition of the in vitro dissolution with the in vivo estimated absorption/dissolution from the pharmacokinetics of the metabolite.

## 4. Discussion

Considering the pharmacokinetics of drugs that undergo substantial metabolism, that would be classified as BDDCS (Biopharmaceutics Drug Disposition Classification System) Class 1 and 2 compounds [35], the following essential sequence should be considered: in vivo dissolution (correlated with the in vitro dissolution), absorption, and metabolism of the parent drug.

**Figure 9:**
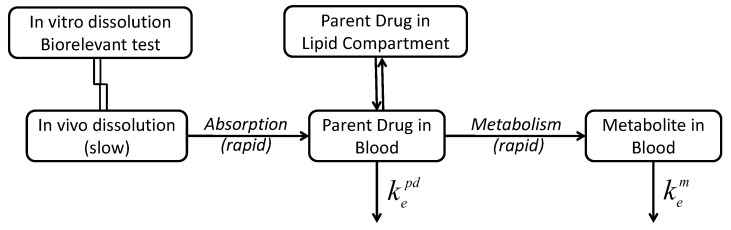
Schematic of processes involved in the pharmacokinetics of drugs that undergo substantial metabolism.

Since the rate and extent of absorption, and the metabolism are usually high, the slowest rate-determining step for the kinetics of entire process remains the release/dissolution of the parent drug from the pharmaceutical formulation. Consequently, the rate of metabolite appearance in the plasma is determined by the rate and extent of parent drug release and dissolution from the pharmaceutical formulation (Figure 9).

**Figure 10:**
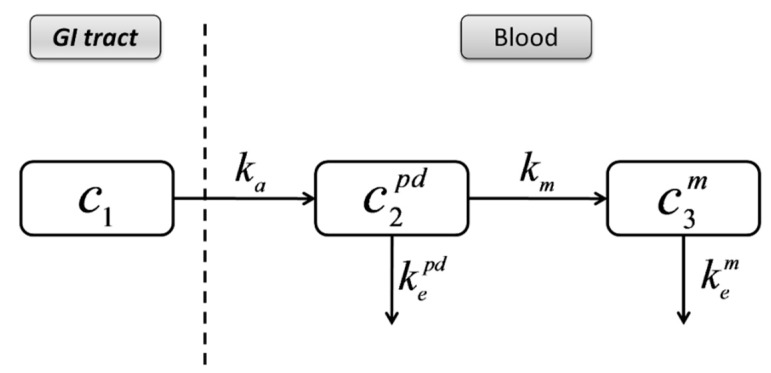
Simplified compartmental model describing the pharmacokinetics of DTZ and DTZM.

Since DTZ is lipophilic (logP 2.79) [31], the transfer rate constant from blood to peripheral compartments is higher than the reverse transport. The rate of return of DTZ to the blood would be small and could be neglected, and the transfer from the blood to the peripheral compartments can be integrated in a total elimination rate constant of the parent drug, $k_{e}^{pd}$ (Figure 10).

If the elimination of the metabolite is not rate limiting (that is when the slowest step is the elimination of the parent drug), the terminal half-life of the metabolite is the same or lower than the terminal half-life of the parent drug. This would be expected, as one of the “objectives” of metabolism is the transformation of drugs in more polar components, for an easier elimination. A one-compartment model can describe the pharmacokinetics of the metabolite, based on the following equation for extravascular administration: $c_{m}\left(T\right)=Ae^{-k_{a}T}+Be^{-k_{e}^{m}T}$. The apparent absorption rate constant $k_{a}$ is a function of the in vivo dissolution, absorption, distribution, metabolism, and elimination of the parent drug, and $k_{e}^{m}$ is the elimination rate constant for the metabolite. Consequently, it is expected that in vitro dissolution of parent drug - in vivo pharmacokinetics of metabolites correlations are possible for these drugs, and that these correlations account for the entire chain of in vivo processes, i.e., dissolution, absorption, metabolism of the parent drug, and direct appearance of the metabolite in the plasma.

The success of the correlation between the in vitro and in vivo dissolution would depend on the in vitro dissolution method. The good correlations between the in vitro dissolution of DTZ and the apparent absorption of DTZ, suggest that the rate determining process is the in vivo dissolution of the parent drug. In this study a successful prediction of DTZ pharmacokinetics after a single dose based on in vitro data only could be achieved.

## 5. Conclusions

Pharmacokinetic modeling of DTZ and DTZM mean plasma levels suggests a one-compartmental behavior. In the case of DTZ and, more generally, in the case of compounds subjected to extensive metabolism (BDDCS Class 1 and Class 2 compounds), the in vivo dissolution, absorption, metabolism of the parent drug, and the elimination of the metabolite would take place. Since the rate of absorption and the metabolism of BDDCS Class 1 and Class 2 compounds drugs are usually high, the rate of the appearance of the metabolites in the plasma is determined by the rate and extent of parent drug release from the pharmaceutical formulation. Under these conditions, a deconvolution method, similar to that of Wagner–Nelson method, can be applied to calculate the absorption and in vivo dissolution of a parent drug starting from the plasma levels of one of its metabolites. The correlation of the estimated in vivo dissolution curves with the in vitro dissolution curves proved to be linear, and in the case of the metabolite a very good superposition of the in vivo and in vitro dissolution kinetics was achieved. Upon further validation with more drugs, this type of correlations could be used for drugs with extensive metabolism, in which the plasma levels of the active metabolites that follow pseudomono-compartmental kinetics are higher than those of the parent drug.

## Figures and Tables

**Table 1:** In vitro dissolution of DTZ from 60 mg Cardiazem^®^ tablets in 900 mL water, using USP Apparatus 2, at 75 rpm (*n* = 12).

Time (min)	Dissolved (%)	CV (%)
10	12.42	10.56
15	17.00	9.04
20	18.67	15.88
30	22.17	11.19
60	37.67	10.46
120	63.58	9.66
180	80.92	8.29

**Table 2:** Accuracy and precision data for the determination of diltiazem (DTZ) and desacetyl DTZ (DTZM) in human plasma.

Sample Code	DTZ				DTZM			
	Nominal conc. (ng/mL)	Measured conc. (Mean ± SD, ng/mL)	RSD (%)	Bias (%)	Nominal conc. (ng/mL)	Measured conc. (Mean ± SD, ng/mL)	RSD (%)	Bias (%)
**Within-run**								
LLOQ	2.5	2.49 ± 0.14	5.71	−0.56	1.25	1.26 ± 0.05	3.79	0.40
QC_low_	7.5	7.48 ± 0.29	3.89	−0.25	3.75	3.72 ± 0.14	3.73	−0.85
QC_med_	150	148.83 ± 2.76	1.85	−0.78	75	72.18 ± 1.65	2.29	−3.76
QC_high_	300	310.46 ± 3.70	1.19	3.49	150	146.26 ± 2.47	1.69	−2.49
**Between runs**								
LLOQ	2.5	2.63 ± 0.17	6.43	5.04	1.25	1.32 ± 0.07	5.32	5.60
QC_low_	7.5	7.52 ± 0.31	4.15	0.24	3.75	3.74 ± 0.30	8.03	−0.30
QC_med_	150	146.22 ± 6.68	4.57	−2.52	75	73.71 ± 3.42	4.64	−1.72
QC_high_	300	298.91 ± 12.52	4.19	−0.36	150	149.37 ± 5.27	3.53	−0.42

**Table 3:** Summary pharmacokinetic parameters of DTZ and DTZM in healthy subjects estimated by non-compartmental analysis.

Parameter	DTZ		DTZM	
	Mean	SD	Mean	SD
C_max_ (ng/mL)	154	59.6	6.66	1.98
T_max_ (h)	2.66	0.898	4	1.12
k_e_ (1/h)	0.157	0.0237	0.074	0.0325
t_1/2_ (h)	4.51	0.655	11.2	5.25

**Table 4:** Pharmacokinetic parameters of DTZ and DTZM estimated by one-compartmental analysis of the mean concentration–time profiles after the oral administration of 120 mg of DTZ to 19 healthy subjects.

Parameter	DTZ	DTZM
k_e_ (1/h)	0.2861	0.0945
k_a_ (1/h)	0.0655	0.492
T_lag_ (h)	0.36	0.162
C_max_ (ng/mL)	120.5	6.04
T_max_ (h)	2.51	4.20
AUC_0–∞_ (ng/mL·h)	800.9	94.66
t_1/2_ (h)	2.42	7.34
